# Proceedings of the Mississippi Valley Association of Dental Surgeons, Third Annual Meeting

**Published:** 1847-10

**Authors:** 


### Dental Register of the West.

VOL. 1.] OCTOBER, 1847. [NO. 1.

*Proceedings of the Third Annual Meeting of the Mississippi Valley Association of Dental Surgeons.*

The third annual meeting of the Mississippi Valley Association of Dental Surgeons, commenced its session in the Lecture room of "The Ohio College of Dental Surgery," in Cincinnati, Tuesday September 7, 1847, at 10 o'clock, A. M. A quorum not being present, the society adjourned to meet at 4 o'clock, P. M.

4 O'CLOCK, P.. M

The society met, and after the calling of the roll, proceeded to organize. The President being prevented from attending by family affliction, Dr. Ide, first Vice President, took the chair.

Reports from Committees were called for by the chair, whereupon Dr. J. Taylor rose and remarked, that a number of the members of the Executive and Examining Committees were absent, and moved that the chair proceed to fill the vacancies in said Committees; which was carried.

The Committees, when the vacancies were filled, stood as follows, viz:

*Examining Committee*—Drs. J. TAYLOR, J. P. ULLERY, and A. BERRY.

*Executive Committee*—Drs. M. ROGERS, J. ALLEN, J. TAYLOR, A. BERRY, and C. BONSALL.

The Treasurer presented his report, which, on motion, was re-

Cincinnati, Ohio.

W1
DE346G

ferred to an auditory Committee, to be appointed by the chair. Drs. Jas. Taylor, J. Allen and A. Berry, were appointed said committee.

The committee on the communication from Prof. Drake to this society, at its last session, reported that they had learned from Prof. Drake, that he had concluded to defer the publication of his proposed medical work for a short time, and the committee, not having their report ready, was continued.

On motion, Dr. Berry proceeded to read an address—in accordance with a resolution passed at the last meeting of this society—on the use of Tobacco, as connected with Dental Hygiene.

Remarks were offered on the subject of Dr. Berry's address, by Drs. Allen, Rogers, and James Taylor, commending in the main the doctrines and views presented by Dr. Berry.

When, on motion, the address was received to be placed among the archives of the society.

On motion, adjourned to meet at half past 7 o'clock, at the office of Drs. Ross and Berry.

Evening Session, 7½ o'clock.

In the absence of the Recording Secretary, Dr. C. Bonsall was elected Secretary pro tem.

The Corresponding Secretary presented his report as follows:

That the Publishing Committee placed in my hands, for distribution, several hundred copies of the minutes of the proceedings of the society, at its last session—nearly all of which has been sent to various members of the profession throughout the country—also to many distinguished literary men, the object being to honorably bring the society before the public, which, alike with the profession, feel an interest in the advance of Dental Science.

According to resolutions of the Society, some three weeks since, notice was given of the present meeting in several of the leading papers of this city, Louisville and Maysville.

Also that several letters have been received from the following members of the society, containing their annual dues, and explaining the cause of non attendance: Drs. B. B. Brown, St. Louis, Joseph Sanford, Chillicothe, Edward Buckwell, Zanesville, Samuel Griffith, Louisville, Joseph Taylor, Maysville, and D. P. Hunt, Indianapolis; which, with this report, is respectfully submitted.

JAMES TAYLOR, Cor. Sec'y.

Which was, on motion, adopted, and ordered to be placed on file.

Letters from Drs. D. P. Hunt, B. B. Brown, Samuel Griffith, E. Buckwell, and Joseph Sanford, were presented by the corresponding Secretary, and ordered to be filed.

The committee to whom was referred the Treasurer's accounts, submitted their report, which, on motion, was accepted, and the committee discharged.

*Auditing Committee's Report.*—That they had examined the Treasurer's account, and find it to be correct.

A report was received from the Executive Committee on the publication of a Quarterly Journal, which elicited an animated discussion. The subject was recommitted with instructions to the committee to make further enquiries in relation to the cost of publishing and report before final adjournment.

Ordered that the sum of $2.00 be paid to the Corresponding Secretary to satisfy a bill for Postage, Stationery, &c.

An address from Dr. Allen on the contour of the face was read, which was accepted. The Dr. exhibited several Daguerreotype likenesses, with and without his apparatus for improving the contour of the face, and gave a description of said apparatus. After considerable discussion on the subject of the Address, it was ordered that it should be placed among the archives of the society.

On motion, adjourned to meet to-morrow at 3 o'clock, P. M., at the Ohio College of Dental Surgery.

WEDNESDAY, SEPT. 8, 3 O'CLOCK, P. M.

The society met pursuant to adjournment, Dr. Edward Taylor, President, in the chair.

The minutes of the preceding meetings were read and approved.

The President then proceeded to read the opening address, which was received and placed on file.

The Committee on the publication of a Dental Periodical, reported that they have obtained statistics with respect to the cost of publishing a quarterly journal, and think the cost entirely within the ability of the society, and those in the profession interested, and we believe willing to aid in supporting such a work, and would therefore advise the publication of such a periodical.

JAS. TAYLOR, *Chairman*,

After considerable discussion on the subject, in which Drs. Bonsall, Berry, James Taylor, Rogers, and Allen participated; on motion of Dr. Berry, it was *Resolved*, That this society proceed to publish a Quarterly Journal, to consist of at least 48 pages; the first issue to number 500, the following 300 copies.

On motion of Dr. Bonsall, *Resolved*, That the Committee of Publication shall consist of *three*, who shall be elected, and shall *edit* and *publish* said Journal.

On motion of Dr. Rogers, the society went into the election of officers for the following year, which resulted as follows, viz:

*Pres't*, Wm. B. Ross.

1*st V. Pres't*, W. E. Ide.

2*d V. Pres't*, D. J. Hunt.

3*d V. Pres*'t, B. B. Brown.

*Recording Secretary*, J. Allen.

*Cor. Secretary*, James Taylor.

*Treasurer*, C. Bonsall.

| | |
|---|---|
| *Examining Com.* | W. H. Goddard,<br>M. Rogers,<br>Edward Taylor. |
| *Executive Com.* | Samuel Griffith,<br>A. Berry,<br>J. Allen. |
| *Editing and Pub'ng Com.* | S. P. Hullihen,<br>B. B. Brown,<br>James Taylor. |

On motion, the sum of one dollar was ordered to be paid to the Janitor of the College. On motion of Dr. Taylor, a vote of thanks was tended to the Recording Secretary for his fidelity in office for the past 3 years.

On motion, adjourned to meet at Dr. Taylor's office, at half past 7 o'clock, P. M.

Evening Session, 7½ o'clock.

The President called the society to order. The minutes of the afternoon were read and approved.

After some discussion on the subject, it was *Resolved*, That the

Journal to be published by this society shall be called the "Dental Register of the West."

An address was read by Dr. Rogers, on "Nerve killing," which was accepted, and orderod to be placed on file.

Dr. J. Taylor then proceeded to read an address on Dental Hygiene, which was also accepted and placed on file.

The following gentlemen were appointed to deliver addresses at the next annual meeting—the President to deliver the opening address, viz:

| | | |
|---|---|---|
| Dr. B. B. Brown, on | | |
| " Wm. M. Hunter, on | | Black Teeth. |
| " H. Crane, | " | Plugging Teeth. |
| " Joseph Sanford, | " | Extracting Teeth. |
| " W. E. Ide, | " | Irregularities of the teeth. |
| " J. W. Cook, | " | Cleanliless of the mouth. |
| " C. Bonsall, | " | Pivot teeth. |

Ordered, that the proceedings of this meeting be published in the first number of the Dental Register.

Minutes read and approved, and the society adjourned to meet on the first Tuesday in September, 1848, at 10 o'clock, A. M., in the Ohio College of Dental Surgery.

E. TAYLOR, *Pres't.*

Wm. B. Ross, *Sec'y.*

---

*Address by* E. Taylor, *M. D. D. D. S., President Mississippi Valley Association of Dental Surgeons; delivered at its Third Annual Meeting.*

